# Lower Local Dynamic Stability and Invariable Orbital Stability in the Activation of Muscle Synergies in Response to Accelerated Walking Speeds

**DOI:** 10.3389/fnhum.2018.00485

**Published:** 2018-12-11

**Authors:** Benio Kibushi, Shota Hagio, Toshio Moritani, Motoki Kouzaki

**Affiliations:** ^1^Laboratory of Neurophysiology, Graduate School of Human and Environmental Studies, Kyoto University, Kyoto, Japan; ^2^Japan Society for the Promotion of Science, Tokyo, Japan; ^3^Graduate School of Education, The University of Tokyo, Tokyo, Japan; ^4^School of Health and Sport Sciences, Chukyo University, Nagoya, Japan

**Keywords:** maximum lyapunov exponents, maximum floquet multipliers, electromyography, non-negative matrix factorization, central nervous system, motor control, nonlinear analysis

## Abstract

In order to achieve flexible and smooth walking, we must accomplish subtasks (e. g., loading response, forward propulsion or swing initiation) within a gait cycle. To evaluate subtasks within a gait cycle, the analysis of muscle synergies may be effective. In the case of walking, extracted sets of muscle synergies characterize muscle patterns that relate to the subtasks within a gait cycle. Although previous studies have reported that the muscle synergies of individuals with disorders reflect impairments, a way to investigate the instability in the activations of muscle synergies themselves has not been proposed. Thus, we investigated the local dynamic stability and orbital stability of activations of muscle synergies across various walking speeds using maximum Lyapunov exponents and maximum Floquet multipliers. We revealed that the local dynamic stability in the activations decreased with accelerated walking speeds. Contrary to the local dynamic stability, the orbital stability of the activations was almost constant across walking speeds. In addition, the increasing rates of maximum Lyapunov exponents were different among the muscle synergies. Therefore, the local dynamic stability in the activations might depend on the requirement of motor output related to the subtasks within a gait cycle. We concluded that the local dynamic stability in the activation of muscle synergies decrease as walking speed accelerates. On the other hand, the orbital stability is sustained across broad walking speeds.

## Introduction

We can easily walk under various conditions; controlling flexible and smooth walking is a necessary factor for daily life. To achieve flexible and smooth walking, we must achieve subtasks within a gait cycle (e.g., loading response, forward propulsion or swing initiation) within a gait cycle (Winter, 1987). Walking is achieved by controlling subtasks at appropriate times within a gait cycle. Dysfunctions in subtasks within a gait cycle are directly associated with impairments in walking ability. For example, elderly adults frequently cause the co-activation between the ankle plantar flexor and the ankle dorsiflexor muscles during the forward propulsion (Schmitz et al., 2009). Moreover, post-stroke individuals with hemiparetic walking induce the co-contraction between the hip flexor and the hip extensor muscles during the stance phase (Den Otter et al., 2007). Because the assessment of walking ability is a critical factor in rehabilitation, effective evaluation of subtasks within a gait cycle may be beneficial for clinicians. Such evaluation of subtasks within a gait cycle may be achieved via analysis of muscle synergies. Many previous studies have revealed that the central nervous system (CNS) may modularly organize movements via the muscle synergies that control several muscles (Tresch et al., 1999; d'Avella et al., 2003; Hagio and Kouzaki, 2014; Hagio et al., 2015; Kubo et al., 2017; Nishida et al., 2017; Kibushi et al., 2018). The activation of muscle synergies may reflect the control signal from the CNS. During walking, sets of extracted muscle synergies characterize muscle patterns that are related to subtasks within a gait cycle (e.g., loading response phase, late stance phase or swing phase; Neptune et al., 2009; McGowan et al., 2010; Allen and Neptune, 2012). In addition to walking, muscle synergies are observed during running, sidestepping, backward walking and perturbed walking (Chvatal and Ting, 2013; Oliveira et al., 2013; Zelik et al., 2014; Yokoyama et al., 2016; Nishida et al., 2017). Thus, muscle synergies characterize coordination patterns of muscles that relate to subtasks in various locomotor behaviors. When the walking speed changes, constructions of muscle synergies are relatively consistent. However, activations of muscle synergies are affected by the walking speed. Our previous study showed that construction of muscle synergies was relatively consistent among walking speeds (Kibushi et al., 2018). On the other hand, the timing of intense activation within one gait cycle of muscle synergies shifts depending on walking speeds. Moreover, peak activation of muscle synergies becomes larger as walking speed increases (Ivanenko et al., 2004; Yokoyama et al., 2016). Hence, activation of muscle synergies depends on walking speed. To evaluate impairments in subtasks within a gait cycle, analysing the activation of muscle synergies may be effective. Allen et al. (2013) revealed that merged temporal patterns of synergies during hemiparetic walking reflect impairments in subtasks within a gait cycle. Although many researchers have investigated how the impairments in the CNS affect muscle synergies, it has not been proposed a way to evaluate instability of activation of muscle synergies themselves. We considered that direct analysis of instability in the activation of muscle synergies might characterize instability of neural input signal from the CNS.

The time-series of joint angle or muscle activity during walking is periodic, and the attractor or limit cycle is constructed from the time-series data during walking (Kuo, 2002; Dingwell and Kang, 2007). Because the activation of muscle synergies during walking is also periodic (Ivanenko et al., 2004; Kibushi et al., 2018), we considered that stability of attractor or limit cycle that produces the activation of muscle synergies may reflect the stability of the control signal from the CNS. Stability of attractor or limit cycle has been investigated in the fields of engineering, robotics, and computer simulation for walking (Bruijn et al., 2011; Huang et al., 2014, 2017). In these stability analyses, the stability of time-series data is evaluated by investigating characteristics of an attractor that is reconstructed in a state space. Recently, the maximum Lyapunov exponent and maximum Floquet multiplier were applied in human walking to evaluate stability of attractor that was produced by the time-series data of walking (Dingwell and Marin, 2006; Dingwell and Kang, 2007; England and Granata, 2007; Kang and Dingwell, 2008; Lockhart and Liu, 2008; Bruijn et al., 2009; Santuz et al., 2018). Maximum Lyapunov exponents quantify how the system's states respond to very small local perturbations continuously (Dingwell and Kang, 2007). On the other hand, maximum Floquet multipliers quantify the tendency of the system's states to return to the periodic limit cycle orbit after small perturbations (Dingwell and Kang, 2007). In summary, local dynamic stability is quantified by the maximum Lyapunov exponents, and orbital stability is quantified by the maximum Floquet multipliers. Thus, maximum Lyapunov exponents and maximum Floquet multipliers are quantify different aspect of dynamical stability, respectively. We expected that stability of attractor that was constructed by activation of muscle synergies by investigating maximum Lyapunov exponents and maximum Floquet multipliers.

Previous studies have shown that large motor outputs are required with accelerated walking speeds. For example, the peak ankle plantar flexion moment or the activation of tibialis anterior and medial gastrocnemius muscles increased with acceleration of walking speed (Warren et al., 2004; Pires et al., 2014). For these requirement for large motor outputs, fast walking cannot be persist for a long time. We considered that requirement for large motor output may cause lower local dynamic stability of activation of muscle synergies. Then, we hypothesized that the maximum Lyapunov exponents of activation of muscle synergies increase with acceleration of walking speed. On the other hand, we can achieve periodic walking among widely walking speed. Hence, we supposed that the orbital stability of activation of muscle synergies may be sustained among the walking speed.

The purpose of this study was identifying the local dynamic stability and orbital stability of activation of muscle synergies that relate to subtasks within a gait cycle among various walking speeds. To achieve this purpose, we investigated the maximum Lyapunov exponents and the maximum Floquet multipliers in activation of muscle synergies among various walking speeds.

## Methods

### Experimental Setup and EMG Procedures

We recruited 10 healthy men (age: 23.3 ± 0.9 years, height: 171.1 ± 3.44 cm, and weight: 64.1 ± 0.63 kg) for this study. Subjects provided written informed consent to participate in the study after receiving a detailed explanation of the purposes, potential benefits, and risks associated with participation. The experimental procedures were conducted in accordance with the Declaration of Helsinki and were approved by the Local Ethics Committee of the Graduate School of Human and Environmental Studies, Kyoto University (Approval number 26-H-22). We instructed the subjects to walk naturally on a treadmill (Adventure 3 PLUS, Horizon, Johnson Health Tech Japan Co., Tokyo, Japan) at 2.0, 2.5, 3.0, 3.5, 4.0, 4.5, 5.0, 5.5, 6.0, 6.5, 7.0, 7.5, and 8.0 km/h, in a randomized order. Subjects walked on the treadmill over 50 gait cycles at each walking speed. We recorded surface electromyograms (EMG) from 12 muscles in the right lower limb and trunk: the gastrocnemius medialis (MG), gastrocnemius lateralis (LG), soleus (SOL), tibialis anterior (TA), vastus lateralis (VL), rectus femoris (RF), biceps femoris (BF), tensor fasciae latae (TFL), adductor longus (AL), gluteus medius (Gmed), gluteus maximus (Gmax), and erector spinae (ERE). The electrode placements were carefully chosen to minimize crosstalk from the adjacent muscles by using an ultrasonic device, and we placed EMG electrodes based on suggestions from SENIAM (seniam.org), the European project on surface EMGs. The EMG signals were amplified (SX230-1000, Biometrics, Gwent, UK) and bandpass filtered between 20 and 450 Hz (Kouzaki and Shinohara, 2010). All electrical signals were stored at a sampling frequency of 1,000 Hz on the hard disk of a personal computer using a 16-bit analog-to-digital converter (PowerLab/16SP; AD Instruments, Sydney, Australia). To record the heel contact timing, subjects wore shoes that were implemented switches. We defined 1 gait cycle as the start of one right heel contact to the moment before the next right heel contact. We analyzed 30 gait cycles within the sampled EMGs from each subject. It has been revealed that the kinematics of initial walking differs from that of steady walking (Mbourou et al., 2003; Bus and de Lange, 2005). Therefore, we excluded the initial 10 gait cycles from analysis. Based on similar reason, we excluded the last 10 gait cycle from analysis. During the experiment, subjects took adequate rest between the tasks (rest time was changed in accordance with situations) to reduce the fatigue. Moreover, time for achieving a gait task is 40–100 s, and all tasks (including rest time) were finished within 1 h. We inquired whether subject felt fatigue during experiment, no subjects claimed fatigue. Therefore, we expect the fatigue did not affect results. Before extraction of the muscle synergies, the EMGs were high-pass filtered (40 Hz) with a zero lag fourth-order Butterworth filter, full-wave rectified, low-pass filtered (10 Hz) with a zero lag fourth-order Butterworth filter, and time-interpolated over 100 points (Cappellini et al., 2006; Clark et al., 2010). Using these procedures, we provided a 12 muscles × 30 gait cycles-sized matrix (12 muscles × 3000 time steps) for each subject. The EMG matrix was normalized to the peak activity of the EMGs for all muscles (Torres-Oviedo and Ting, 2007; Hagio and Kouzaki, 2014). After this normalization, the EMG matrix was normalized to the standard deviation of each muscle to have unit variance (Torres-Oviedo and Ting, 2007; Hagio and Kouzaki, 2014).

### Extraction of the Muscle Synergy

The muscle synergies were extracted by using a non-negative matrix factorization (NMF) algorithm (Lee and Seung, 1999; Cheung et al., 2005). The NMF approximately decomposes a matrix into two non-negative matrixes by minimizing an error between the original matrix and a reconstructed matrix. The particular muscle activation pattern for a given walking speed is represented by the following equation:

(1) $$\begin{array}{r} \begin{array}{c} M\;=\sum_{i=1}^{N}{W_{i}\;C}_{i}+\varepsilon \;W_{i}\geq 0\;C_{i}\geq 0 \end{array} \end{array}$$

where ***N*** is the number of synergy, ***W***_***i***_ is the muscle weighting in a muscle synergy *i*, ***C***_***i***_ denotes an activation that involves a relative contribution of the muscle synergy, **ε** is the residual. ***W***_***i***_ is 12 × 1 vector (number of muscles × number of synergies), and ***C***_***i***_ is 1 × 3000 vector (number of synergies × phase points). Each component of ***W***_***i***_ represents the contribution of one particular muscle to that muscle synergy, and an individual muscle may contribute to multiple muscle synergies. The composition of the muscle synergy ***W***_***i***_ does not change within a walking speed, but the activation ***C***_***i***_ does change within a walking speed. The weighting of each muscle synergy and activation coefficient were normalized, such that the individual muscle weighting vector was a unit vector.

### Selection of the Number of Muscle Synergies

**Figure 1:**
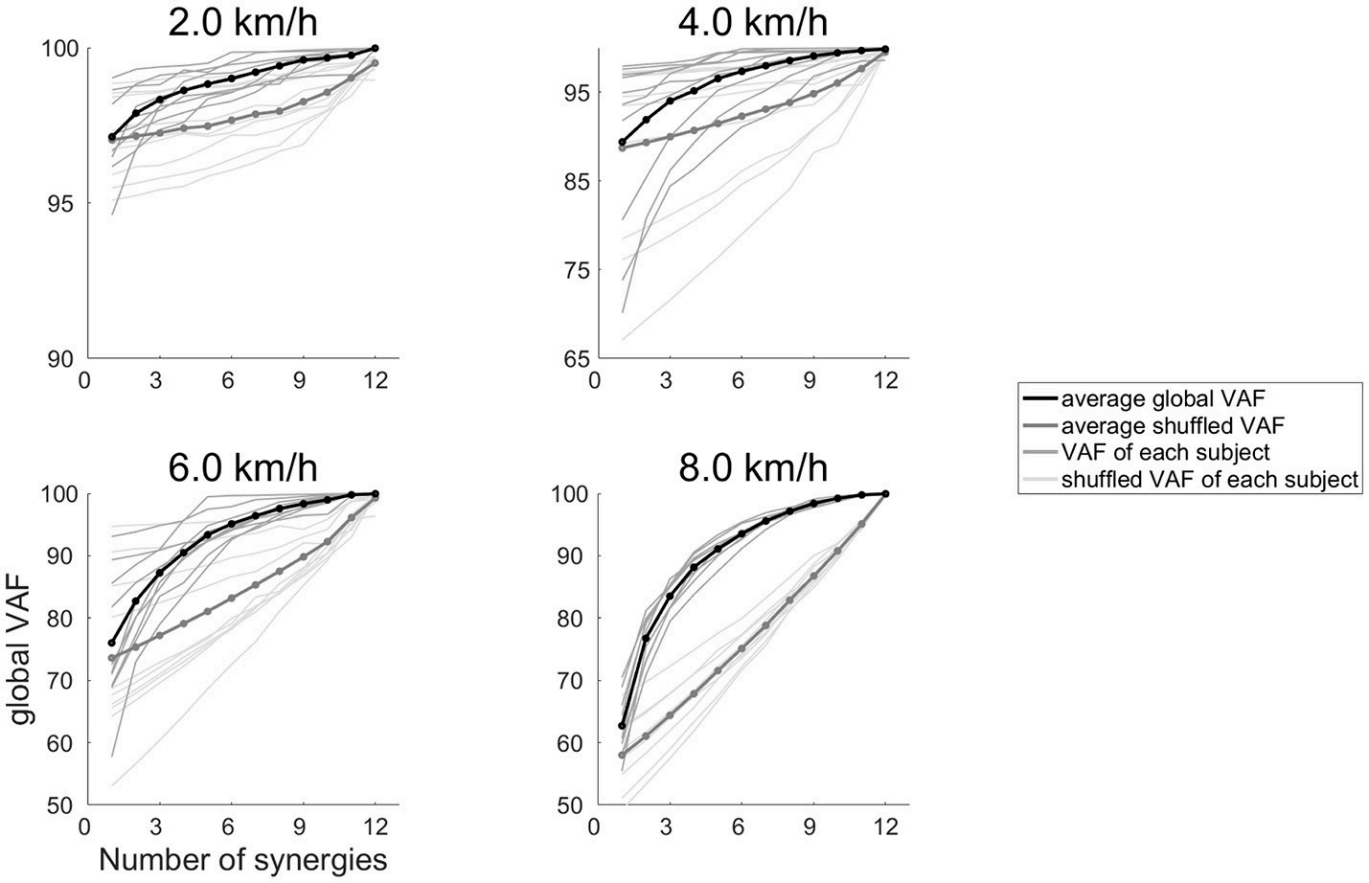
The global VAF values at 2.0, 4.0, 6.0, and 8.0 km/h. The average global VAF values across subjects are illustrated as black lines, and dark gray lines denote the global VAF value for each subject. Thick gray lines represent the average shuffled VAF across subjects, and thin gray lines denotes the shuffled VAF for each subject.

We extracted the muscle synergy and activation coefficient from the EMG data matrix for each walking speed. The muscle synergies were extracted between 1 and 12. We performed cross-validation to obtain consistent muscle synergies. Construction or activation of muscle synergies are slightly different among repetition of extraction, because results by the NMF depend on initial states. In addition, we need to extract invariant muscle synergies within a walking speed. This concept based on the assumption that muscle synergies invariant among gait cycles. Therefore, we considered that the cross-validation would support extracting consistent muscle synergies. To perform cross-validation, we divided the EMG data (30 gait cycles) into 60% of the EMG (18 gait cycles) data, and 40% of the EMG (12 gait cycles) data from each walking speed (Torres-Oviedo and Ting, 2007). The muscle synergies were extracted from 60% of the EMG data to 40% of the EMG data. For updating the weightings and activations in 40% of the EMG data, the activations of the muscle synergies were updated, whereas the weightings of the muscle synergies were fixed by the weightings of 60% of the EMG data (Cheung et al., 2005; Torres-Oviedo and Ting, 2007). This cross-validation was repeated 10 times (Kibushi et al., 2018). In order to determine the appropriate number of muscle synergies, we verified the goodness of fit between the original and reconstructed EMG data matrixes. The original EMG data denotes filtered and normalized EMG data, and reconstructed EMG data indicates the EMG matrix that was reconstructed by Σ***W***_***i***_***C***_***i***_. We defined the number of muscle synergies as the smallest number of synergies that resulted in an adequate reconstruction of the original EMG data. The variability accounted for (VAF) was calculated as a coefficient of determination, which was based on the entire dataset (global VAF) and each muscle (muscle VAF) for each subject (Torres-Oviedo et al., 2006; Hagio and Kouzaki, 2014). The VAF was calculated using the following equation:

(2) $$VAF=[1-(EMG_{o}-EMG_{r}{)}^{2}/EMG_{o}{}^{2}]\times 100$$

where *EMG*_*o*_ represents an EMG data matrix before performing the NMF, and *EMG*_*r*_ denotes the EMG matrix that was reconstructed by Σ***W***_***i***_***C***_***i***_. The term of ***(EMG***_***o***_ **–** ***EMG***_***r***_***)***^**2**^ and $EMG_{o}^{2}$ were the sum of squared. The global VAF and the VAF of each of the 12 muscles were calculated in 40% of the EMG data. The global VAF matrix and 12 muscle VAF matrixes were averaged across repetitions. In addition, the 95% confidence interval (CI) for the VAF matrix at each synergy number (1–12) was calculated. The synergy number (1–12) indicates that synergy number ranged 1–12 because we calculated variability accounted for (VAF) at all number of muscle synergies (ranges 1–12). The number of synergies underlying each dataset was defined as the minimum number of synergies at which the lower bound of the 95% CI exceeded 90% of the global VAF and 75% of the muscle VAF (Clark et al., 2010; Hagio and Kouzaki, 2014; Sawers et al., 2015). We rounded the median number of synergies across subjects, and we determined that the number of synergies were 4 (2.0–4.0 km/h) and 5 (4.5–8.0 km/h). To confirm that the muscle synergies extracted by the NMF algorithm were due to the inherent organization of the muscle activation based on neurophysiological evidence rather than on artifacts produced by the NMF method, the VAF levels were compared to the VAF values of the muscle synergies that were extracted from the shuffled datasets. For the shuffled procedure, the data for each muscle were shuffled independently; therefore, this shuffled data matrix contained the same values, range and variance for each muscle, whereas the relationships among the muscle activations were removed (Chvatal and Ting, 2013; Hagio and Kouzaki, 2014). The results of the VAF and shuffled VAF are shown in Figure 1. In all cases, the VAF values for the reconstruction of the original data using the identified muscle synergies were clearly higher than the VAF of the shuffled datasets.

### Calculation of Maximum Lyapunov Exponents and Maximum Floquet Multipliers

We calculated the maximum Lyapunov exponents and the maximum Floquet multipliers of the activations of the muscle synergies. To calculate the maximum Lyapunov exponents and maximum Floquet multipliers, we selected a time-delayed coordinate approach (Dingwell and Cusumano, 2000; Dingwell and Marin, 2006; Dingwell and Kang, 2007; Labini et al., 2012). In the construction of the time-delayed coordinate system, the state space was constructed from single-dimensional time series measurements and its time-delayed copies. The embedding theorem of Takens (1981) ensures the validity of the time-delayed coordinate approach. We constructed the state space from the activation of muscle synergies. The number of data point of the activation of muscle synergies were 3,000. The general form of the state space is:

(3) $$S\left(t\right)=\left[q\left(t\right),q\left(t+\tau \right),\ldots ,q(t+(d_{E}-1)\tau )\right]$$

where *S*(*t*) is the state space, *q*(*t*) denotes the original single-dimensional data, τ is the selected time delay, and *d*_*E*_ is the embedding dimension. It has been recommended that the embedding dimension be unified across the subjects (van Schooten et al., 2013). Therefore, we unified the embedding dimension of the activation of each muscle synergy. To determine the unified embedding dimension, we searched the appropriate embedding dimensions in each measurement by using a Global False Nearest Neighbor (FNN) analysis (Kennel et al., 1992). We determined the embedding dimension when the number of false neighbors on the reconstructed trajectory were minimized. The calculated embedding dimensions were averaged across walking speeds, subjects and activations of the muscle synergies. As a result, we defined the embedding dimension in the activation of the muscle synergies as 6. We determined the time delays by calculating the first minimum of the average mutual information function (Fraser and Swinney, 1986). The time delays of the activations of the muscle synergies were 23 ± 2.1, 23 ± 1.9, 20 ± 2.6, 21 ± 2.9, and 20 ± 2.5% of gait cycle, respectively.

We were able to quantify the average exponential rate of divergence of neighboring trajectories in the state space using the maximum Lyapunov exponent (Rosenstein et al., 1993). The maximum Lyapunov exponent (λ_1_) for a dynamical system can be defined using:

(4) $$\begin{array}{r} {d\left(t\right)=De}^{\lambda_{1}t} \end{array}$$

where *d(t)* is the mean displacement between neighboring trajectories in state space at time *t, D* is the initial separation between neighboring points. The finite-time Lyapunov exponents are distinguished from true Lyapunov exponents (λ_1_), which are strictly defined only in the dual limit as *D* → 0 and *t* → ∞ in (4). The true Lyapunov exponents cannot be computed reliably because it is difficult to approach limits in the experimental data. Then, Rosenstein et al. (1993) provided the method that estimates of finite-time Lyapunov exponents (λ^*^) for each embedded time series. Taking the log transform of both sides of (4), λ^*^ was defined from

(5) $$\begin{array}{r} \begin{array}{c} \ln\left[d_{j}\left(i\right)\right]\approx \lambda^{*}\left(i\Delta t\right)+\ln\left[D_{j}\right] \end{array} \end{array}$$

where *d*_*j*_*(i)* is the Euclidean distance between the *j*th pair of nearest neighbors after *i* discrete time steps. Euclidean distances between neighboring trajectories in state space were calculated as a function of time and averaged over all original pairs of nearest neighbors. The λ^*^ were estimated from the slopes of linear fits to curves defined by

(6) $$\begin{array}{r} \lambda \left(i\right)=\frac{\langle ln\left[D_{j}\left(i\right)\right]\rangle }{\Delta t} \end{array}$$

where *D*_*j*_(*i*) is the Euclidean distance between the *j*th pair of nearest neighbors after *i* discrete time steps, *t* is the sampling period of the time series data and 〈ln[***D***_***j***_(***i***)]〉 denotes the average of ln[***D***_***j***_(***i***)] across all values of *j*. Maximum Lyapunov exponents were estimated from the slopes of the linear fits to the curve. We defined the maximum Lyapunov exponent from the slopes of the linear fits to the divergence curve between 0 and 1 stride (Dingwell and Marin, 2006). When a maximum Lyapunov exponent is negative, the analyzed attractor is stable. On the other hand, the analyzed attractor is unstable when a maximum Lyapunov exponent is positive.

The maximum Floquet multipliers were calculated as eigenvalues of the Jacobian of the Poincaré map. A set of points that are generated by passing through the section of an attractor is called the Poincaré map. We defined the Poincaré map at each percent of the gait cycle (1–100%) (Kang and Dingwell, 2009). Therefore, 100 Poincaré maps were defined for each walking speed. The state space *S*_*k*_ for each gait cycle *k* at that Poincaré section evolved to a state in the following gait cycles *S*_*k*+1_. This was according to the Poincaré map:

(7) $$\begin{array}{r} \begin{array}{c} S_{k+1}=F\left(S_{k}\right) \end{array} \end{array}$$

We defined the limit cycle trajectory as the average trajectory across all strides within a walking speed. The limit cycle trajectory produces a single fixed point S^*^ in each Poincaré map.

(8) $$\begin{array}{r} \begin{array}{c} S^{*}=F\left(S^{*}\right) \end{array} \end{array}$$

For our walking data, we defined the fixed points at each Poincaré map by the average trajectory across all strides within a walking speed. The maximum Floquet multiplier that estimated the effects of small perturbations away from the fixed points was calculated by using a linearized approximation

(9) $$\begin{array}{r} \begin{array}{c} \left[S_{k+1}-S^{*}\right]\approx J\left(S^{*}\right)\left[S_{k}-S^{*}\right] \end{array} \end{array}$$

where *J*(S^*^) defined the Jacobian matrix for the system at each Poincaré section. The Floquet multipliers are the eigenvalues of *J*(S^*^). Any deviation away from the fixed point is multiplied by the Floquet multiplier from the subsequent cycle. Thus, for a limit cycle to be orbitally stable, these complexly valued Floquet multiplier must have a magnitude <1.

Now, we summarize analysis of maximum Lyapunov exponents and maximum Floquet multipliers. For local dynamic stability, we defined maximum Lyapunov exponents by evaluating values of the slopes of linear fits to the divergence curve between 0 and 1 strides. This is called short-term maximum Lyapunov exponents in gait analysis. For orbital stability, maximum Floquet multipliers by calculating the eigenvalues derived from the Jacobian of the Poincaré map. The Floquet multipliers and Lyapunov exponents exhibit the same concept of stability, if a maximum Lyapunov exponent is estimated by adequately long time-series of divergence curve and its linear fitting is valid. However, we defined maximum Lyapunov exponents for “short” time-series (0–1 strides) of divergence curve. Hence, the short-term maximum Lyapunov exponents and maximum Floquet multipliers might exhibit discrepant stability state simultaneously in our analysis (e.g., unstable state of local dynamic stability and stable state of orbital stability).

### Statistics

A one-way repeated measures ANOVA was used to test whether outcome measures of interest were significantly influenced by a change in walking speeds. Tukey's *post hoc* analysis was used when the ANOVA indicated a significant main effect. To investigate changes in the increasing rates of the maximum Lyapunov exponents between slower walking speeds (2.0–5.0 km/h) and faster walking speeds (5.5–8.0 km/h), we verified the significance level of the increasing rate of the maximum Lyapunov exponents using Student's paired *t*-tests. It has been revealed that the energy cost during walking is minimized around the preferred walking speed (Cavagna et al., 1963). We expected that changes of maximum Lyapunov exponents and maximum Floquet multipliers also different between slower walking and faster walking. Before performing Student's paired *t*-test, we used the Shapiro-Wilk test to evaluate whether the data were normally distributed. All results from this test for the data (*P* > 0.05) show that the null hypothesis (sample is taken from a population with normal distribution) should not be rejected; therefore these data are normally distributed. The significance level was set at *p* = 0.05.

## Results

### Characteristics of Muscle Activity

**Figure 2:**
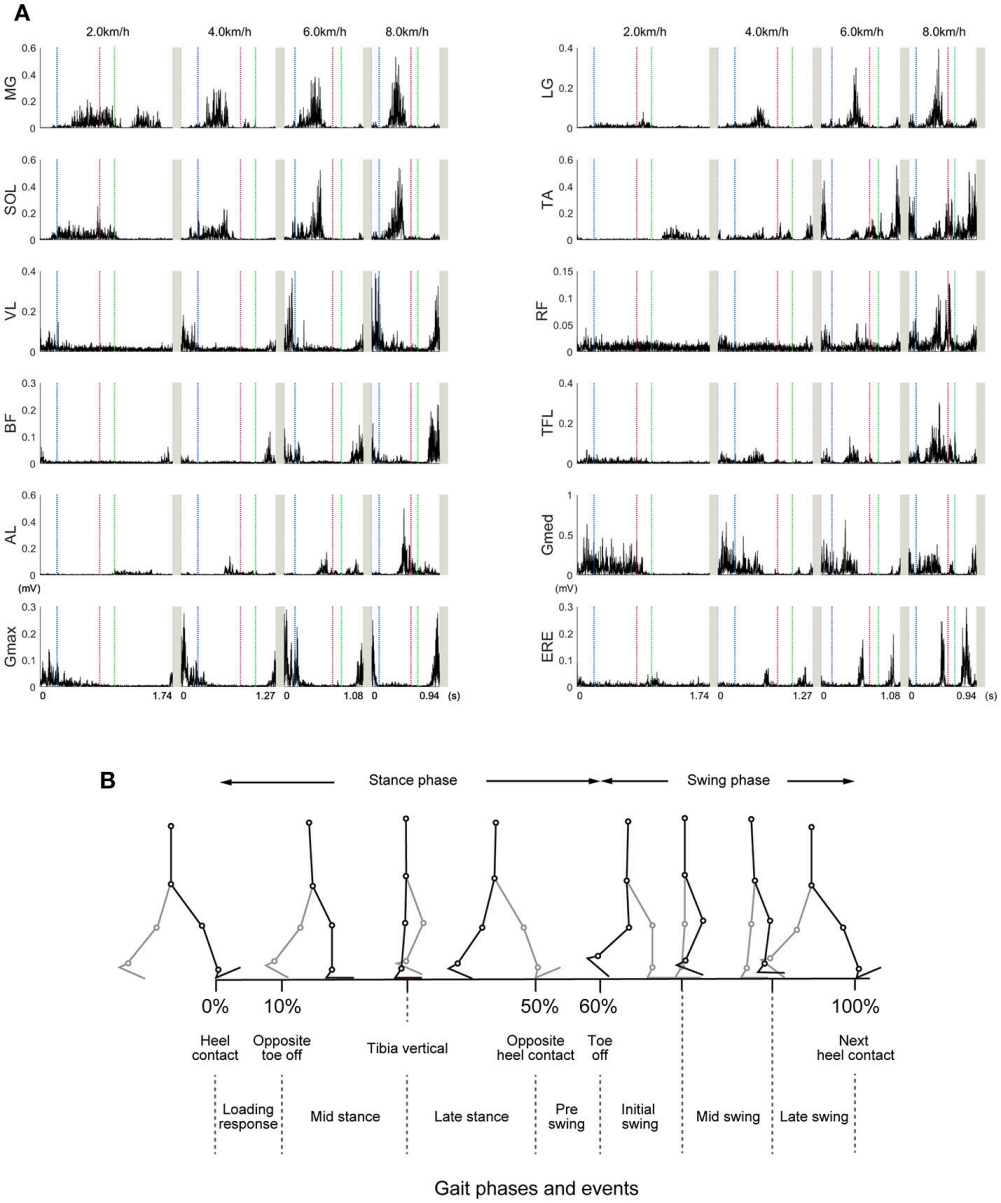
Example time courses of the muscle activities at 2.0, 4.0, 6.0, and 8.0 km/h. **(A)** Representative examples (selected one subject) of the muscle activities within one gait cycle are illustrated. These EMGs were high-pass filtered at 40 Hz, rectified, and demeaned. Vertical dotted lines represent start or end of the gait cycle. The horizontal axis values were based on the time for achieving one gait cycle in representative subject. Blue, red, and green dotted lines indicate a timing of left toe off, left heel contact, and right toe off, respectively. In normal walking, timing of left toe off, left heel contact, and right toe off roughly correspond to 10, 50, and 60% of gait cycle, respectively. The abbreviations represent the gastrocnemius medialis (MG), gastrocnemius lateralis (LG), soleus (SOL), tibialis anterior (TA), vastus lateralis (VL), rectus femoris (RF), biceps femoris (BF), tensor fasciae latae (TFL), adductor longus (AL), gluteus medius (Gmed), gluteus maximus (Gmax), and erector spinae (ERE). **(B)** Different walking phases are shown with stick-pictures. In this figure, one gait cycle is defined from right heel contact to the next right heel contact. Right legs are colored black, and left legs are colored gray.

We measured electromyograms (EMG) from the right limb and trunk muscles. The time series of muscle activity is illustrated in Figure 2A, and illustration of walking phases is shown in Figure 2B. Timing of muscle activation within one gait cycle or changes of muscle activities were consistent with previous reports (Neumann, 2002). The ankle plantar flexors (MG, LG, SOL) were mainly activated during the propulsion phase (40% of gait cycle), and their peak activation increased as the walking speeds became faster. This results consistent with previous studies (Warren et al., 2004). The ankle dorsi-flexor (TA) was mainly activated from just before the heel contact phase to the loading response (10% of gait cycle). At slow walking speeds, the TA inactivated during the loading response. At fast walking speeds, the TA was exceedingly activated during the swing phase (60–100% of gait cycle). The knee extensor (VL) was mainly activated during the loading response. The bi-articular muscle that connects with the knee and hip joint (RF) was activated during the loading response and swing phase. Although the activation of the RF was small at slow walking speeds, muscle activation during the pre-swing phase (around 50% of gait cycle) was considerably high at fast walking speeds. This intensive activity of RF during fast walking speeds was reported in previous studies (Prilutsky and Gregor, 2001). The biceps femoris was activated from just before heel contact and until the loading response. The hip flexor muscle (TFL) was mainly activated during the single support phase (about 10–40% of gait cycle) at slow and moderate walking speeds. At fast walking speeds, the activation of the TFL during the pre-swing phase was extremely high. The hip adductor muscle (AL) was mainly activated during the double support phase (around 50% of gait cycle) until the swing initiation (60% of gait cycle). The hip abductor muscle (Gmed) was mainly activated during the single support phase. The activity of the hip extensor muscle (Gmax) was similar to that of the BF. The trunk stabilizer muscle (ERE) was mainly activated in the double support phase and the post-swing phase (80–100% of gait cycle).

### Extracted Muscle Synergies

**Figure 3:**
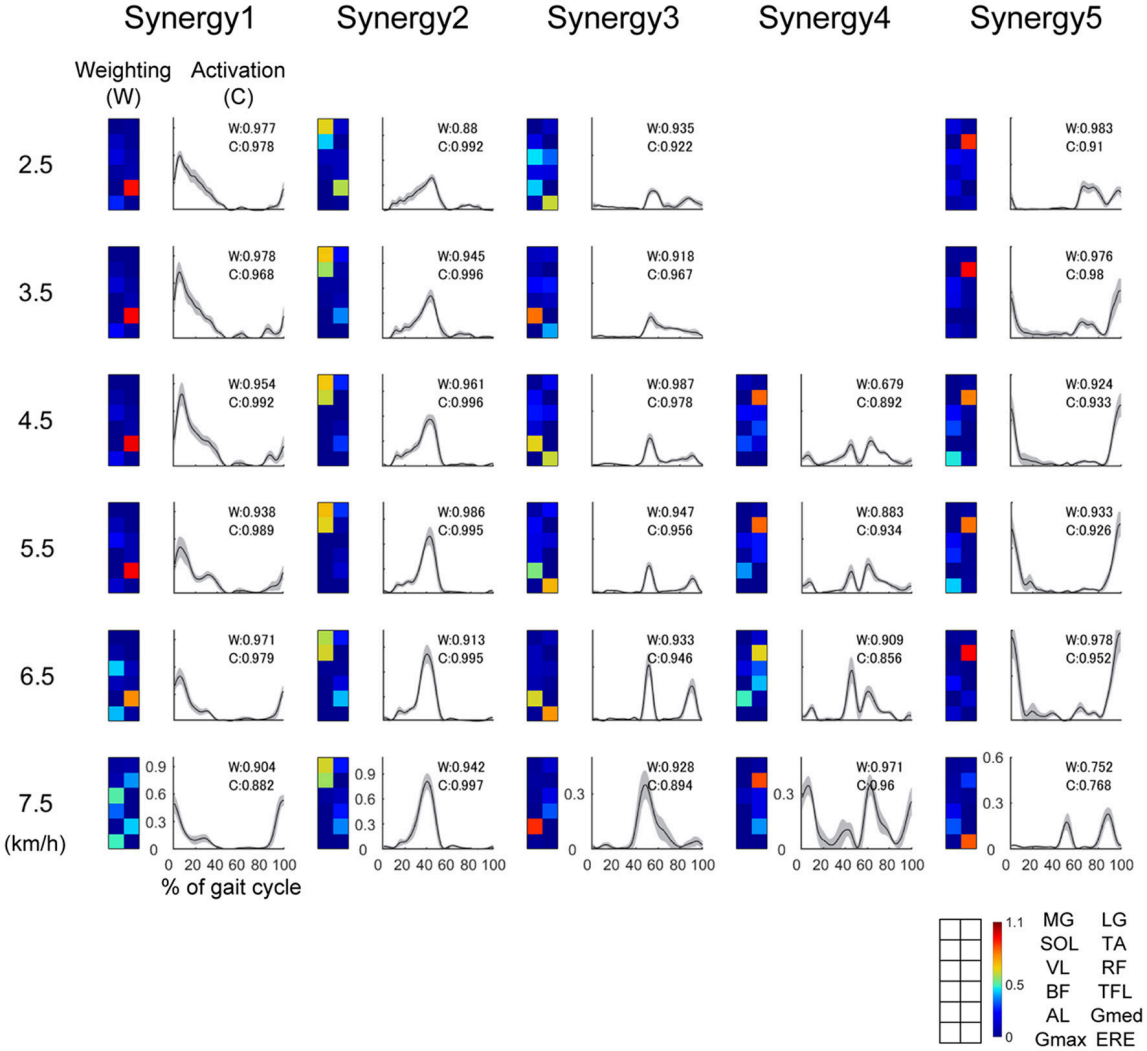
Representative muscle synergies and their activations at 2.5, 3.5, 4.5, 6.7, and 7.5 km/h. The weightings of the muscle synergies (W) are shown as colormaps. Warm color indicates high weightings, and cold color is low weightings. Correspondence between colormap and muscles is described in the lower right of this figure. The activations of the muscle synergies (C) within one gait cycle are shown as waveforms. The horizontal axis indicates % of gait cycle, and vertical axis means amplitude of activation. Black lines denote the average activation over 30 gait cycles, and the gray-shaded area denotes the standard deviation of the activation. We performed functional sorting of the muscle synergies by using cosine similarity (Hagio et al., 2015; Kibushi et al., 2018). Initial functional sorting was performed by grouping the muscle synergies based on the values of cosine similarity compared to that of an arbitrary reference subject. When cosine similarities of W or C were over 0.71 (*p* < 0.01), the muscle synergies were sorted as similar muscle synergies. If two muscle synergies within one walking speed were grouped into the same muscle synergy group, we defined a pair of muscle synergies with the highest correlation as the same group of muscle synergies (Torres-Oviedo and Ting, 2007; Hagio et al., 2015; Kibushi et al., 2018). After initial sorting, we averaged muscle synergies among subjects and those muscle synergies were sorted again. In this second sorting, reference muscles synergies were set as average muscle synergies. The values of cosine similarities between average muscle synergies and other muscle synergies were indicated at right upper side of plots in the activation. Average muscle synergies were represented in Supplementary Figure 1.

Typical examples of the extracted muscle synergies are illustrated in Figure 3. The ***W***_***i***_ is the muscle weighting in a muscle synergy *i*, the ***C***_***i***_ denotes an activation that involves a relative contribution of the *i* th muscle synergy. The weighting of Synergy1 (W_1_) mainly consisted of the knee extensor (VL), hip adductor (Gmed) and hip extensor (Gmax), which were recruited during the single support phase (10–50% of gait cycle) for the loading response (0–10% of gait cycle) and body support. The activation of Synergy1 (C_1_) contributed to body support. The weighting of Synergy2 (W_2_) dominated the plantar flexors (MG, LG, SOL), and main peak activation of Synergy2 (C_2_) was located in forward propulsion phase (40% of gait cycle). Therefore, Synergy2 contributed to the generation of forward propulsion. The weighting of Synergy3 (W_3_) was constructed by the hip abductor (AL), hip flexor (TFL) and trunk stabilizer (ERE) muscles. The activation of Synergy3 (C_3_) was related to the swing initiation and acceleration of the swing leg. The weighting of Synergy4 (W_4_) mainly dominated the ankle dorsiflexor (TA) and hip flexor (RF) muscles, and Synergy4 were activated during the mid-swing phase (around 85% of gait cycle). The activation of Synergy4 (C_4_) was related to the swing leg. Synergy4 was absent between 2.0 and 4.0 km/h. The weighting of Synergy5 (W_5_) mainly consisted of the ankle dorsiflexor (TA) and hip extensor (BF) muscles. This synergy was recruited during the late-swing phase (around 80–100% of gait cycle) to perform ankle dorsiflexion and to decelerate the swing of the leg. The activation of Synergy5 (C_5_) related to the late swing phase.

### The Maximum Lyapunov Exponent and Maximum Floquet Multiplier During Activation of the Muscle Synergy

**Figure 4:**
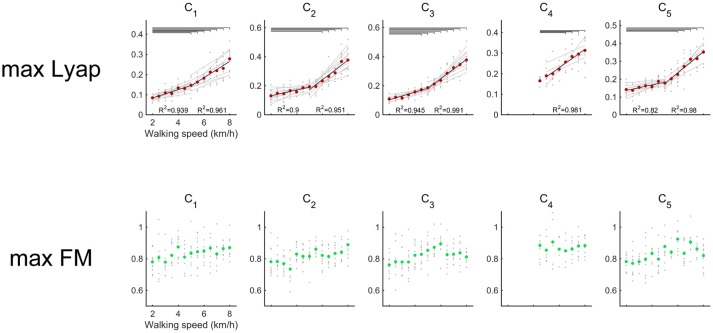
Maximum Lyapunov exponents (upper red) and maximum Floquet multipliers (below green) of the activations of muscle synergy. Gray circles indicate the results of maximum Lyapunov exponents in each subject. Red circles denote the average maximum Lyapunov exponents across all subjects. Black lines represent the average regression lines across the subjects, and gray lines indicate the regression lines for each subject. To detect differences in the increasing rates between slower (2.0–5.0 km/h) and faster walking speeds (5.0–8.0 km/h), the average maximum Lyapunov exponents were regressed separately. The results of *R*^2^ are shown in the lower side of each plot. The left *R*^2^ represents slower walking speeds, and the right *R*^2^ represents faster walking speeds. Upper horizontal lines denote significant differences in the maximum Lyapunov exponents *(p* < 0.01). Green circles show the average maximum Floquet multipliers across the subjects.

In all of the activations, the maximum Lyapunov exponents were positive (Figure 4). It means that the attractor in activations of the muscle synergies indicate local instability. We compared differences in the maximum Lyapunov exponents among walking speeds (Figure 4). The maximum Lyapunov exponents of the activation that contributed to body support (C_1_) and the activation that dominated the late swing phase (C_5_) at 2.0 km/h were significantly smaller than the Lyapunov exponents over 6.0 km/h (*p* < 0.01). Significant differences in the maximum Lyapunov exponents were observed between 2.0 km/h and 5.0 km/h in the activation that generated forward propulsion (C_2_) (*p* < 0.01). The maximum Lyapunov exponents in C_2_ gradually increased at slow and moderate walking speeds, and the maximum Lyapunov exponents in the C_2_ considerably increased at fast walking speeds. In the activation that related to the swing phase (C_3_), significant differences were shown between 2.0 and over 4.5 km/h (*p* < 0.01). As we mentioned in the Introduction section, fast walking speeds require large motor outputs. Therefore, the requirement for large motor output that was due to the fast walking speeds may be a main factor in the high maximum Lyapunov exponents of activations.

Maximum Floquet multipliers of the activations were illustrated in Figure 4. The averaged maximum Floquet multipliers in the muscle synergies were >1. We verified significant difference of maximum Floquet multipliers in the activations among walking speeds. Although the maximum Lyapunov exponents in the muscle synergies increased as walking speeds got faster, the maximum Floquet multipliers were relatively consistent with the changes in walking speed (*p* > 0.05).

**Figure 5:**
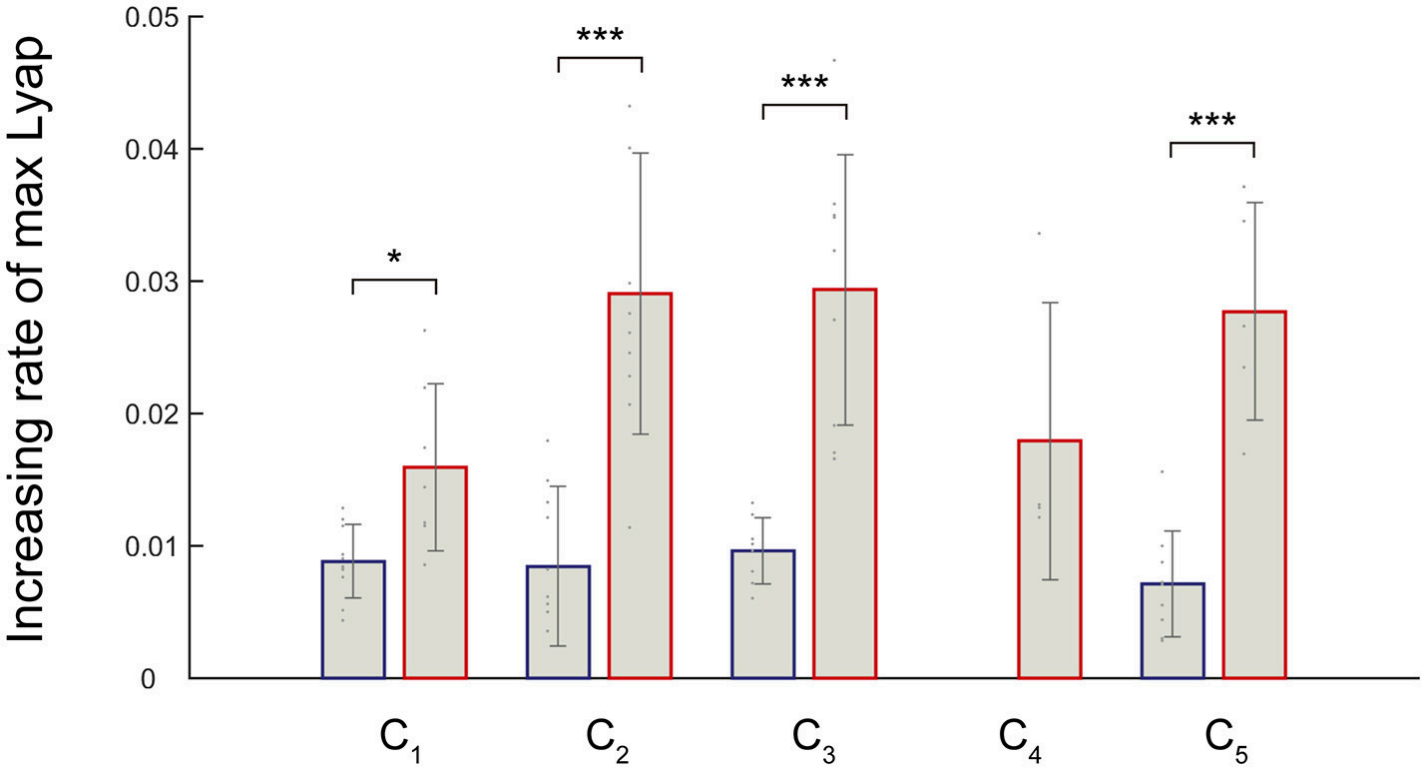
Increasing rate of the maximum Lyapunov exponents in response to the activation of a muscle synergy. Blue bars denote the average increasing rate of the maximum Lyapunov exponents at slower walking speeds (2.0–5.0 km/h). Red bars represent average the increasing rate of the maximum Lyapunov exponents during faster walking speeds (5.0–8.0 km/h). Error bars denote standard deviations among subjects. Significance levels are illustrated as asterisks (^*^p < 0.05, ^***^p < 0.01).

We noticed that an increasing rate of maximum Lyapunov exponents were high among the fast walking speeds. Therefore, we verified the significance level of the increasing rate of the maximum Lyapunov exponents. We expected that the differences in the local dynamic stability in the muscle synergies could be observed in increasing rate of maximum Lyapunov exponents. We compared the increasing rate of maximum Lyapunov exponents between slower walking speeds (2.0–5.0 km/h) and faster walking speeds (5.0–8.0 km/h) (Figure 5). Because synergy4, which contributed to swing initiation (60% of gait cycle), was absent between 2.0 and 4.0 km/h, we did not calculate the increasing rate of maximum Lyapunov exponent at the slower walking speeds (2.0–5.0 km/h) in the C_4_. As a result, we found that the increasing rate in the maximum Lyapunov exponents at faster walking speeds were significantly higher than slower walking speeds (*p* < 0.05). This result indicated that the maximum Lyapunov exponents in activations rapidly increased at fast walking speeds. In addition, we found that the increasing rate of the C_1_, which related to the loading response, at faster walking speeds was relatively low. This implied that the local dynamic stability decreased almost linearly in the C_1_ only. Other maximum Lyapunov exponents in the activation of muscle synergies C_2_ (forward propulsion), C_3_ (swing phase), and C_5_ (leg deceleration) rapidly increased at faster walking speeds. This difference in the increasing rate of maximum Lyapunov exponents might have been associated with differences in the requirements for motor output that related to subtasks within a gait cycle.

## Discussion

To identify the local dynamic stability and orbital stability of the activation of the muscle synergies, we investigated the maximum Lyapunov exponents and maximum Floquet multipliers across various walking speeds. We revealed that the maximum Lyapunov exponents of the activations were positive, and they increased with accelerated walking speeds. Moreover, we found a difference in the increasing rate of the maximum Lyapunov exponents among the muscle synergies that related to subtasks within a gait cycle. Contrary to the maximum Lyapunov exponents, the maximum Floquet multipliers of the activations were almost constant across walking speeds.

Although we found slight differences among the composition of the muscle synergies and the analyzed number of gait cycles, the number of synergies and functions of the muscle synergies were similar to the muscle synergies that were extracted in previous studies (Ivanenko et al., 2004; Neptune et al., 2009; Chvatal and Ting, 2013). We observed that the maximum Lyapunov exponents of the activations increased with accelerated walking speeds (Figure 4). In contrast, the maximum Floquet multipliers of the activation of muscle synergies did not depend on walking speeds. These results indicated that the maximum Lyapunov exponents among the activations of the muscle synergies was high; on the other hand, the maximum Floquet multipliers of the activations of the muscle synergies was sustained during walking. If the dynamical system based on the deterministic system, the maximum Lyapunov exponents should be same among different variables. However, our results did not exhibit same maximum Lyapunov exponents among walking speeds. This may indicate that the dynamical system during walking is constructed by some different systems. In the previous studies, the maximum Lyapunov exponents and maximum Floquet multipliers of experimental data (joint angle or muscle activities) during walking were investigated, and it was reported that maximum Lyapunov exponents were positive although the maximum Floquet multipliers were >1 (Hurmuzlu and Basdogan, 1994; Ali and Menzinger, 1999; Dingwell and Kang, 2007; Su and Dingwell, 2007). This means that attractors during walking can simultaneously exhibit both locally stable and locally unstable regions and still remain orbitally stable (Ali and Menzinger, 1999; Dingwell and Kang, 2007). In addition, Hausdorff et al. (1995) proposed that the variability of stride intervals during walking exhibit aperiodic fractallike fluctuations. Thus, attractor or limit cycle that was produced by experimental walking data may be influenced by some different systems. We suggested that different maximum Lyapunov exponents were observed in this study because different system affected producing the attractor or limit cycle.

We supposed that walking speed may be an important factor for the local dynamic stability and orbital stability in the activations of the muscle synergies. Therefore, we mainly discussed the speed-dependent changes that affected the maximum Lyapunov exponents and maximum Floquet multipliers of the activations.

### The Maximum Lyapunov Exponents in the Activation of Muscle Synergies During Fast Walking Speeds

We extracted the muscle synergies that related to the loading response (Synergy1) (Figure 3). Synergy1 mainly comprised the VL, Gmed and Gmax. It has been reported that the muscles of the VL, Gmed and Gmax contribute to the loading response (Liu et al., 2008; Neptune et al., 2008; Correa et al., 2010; Allen and Neptune, 2012). In addition, the peak knee flexion moment and peak knee power absorption of the loading response increased (Lelas et al., 2003). Thus, the output of the loading response increased with the acceleration in walking speeds. We considered that the requirement of large loading responses might have affected the increasing maximum Lyapunov exponents in the activations related to the loading response (C_1_).

The maximum Lyapunov exponents in Synergy2, which mainly comprised the ankle plantar flexor muscles (MG, LG, SOL), were higher at faster walking speeds (5.0–8.0 km/h) than those at slower walking speeds (2.0–5.0 km/h) (Figure 5). At normal walking speeds, the ankle plantar flexors perform near-isometric contractions during the stance phase (0–60% of gait cycle) via near-optimal fascicular length; this indicated that the muscle work required for stance is extremely small (Fukunaga et al., 2001; Lichtwark et al., 2007; Arnold et al., 2013). This energy-saving during stance contributes to the efficiency of walking. However, fascicular length shortened at fast walking speeds, and the fascicle shortening velocities increased (Arnold et al., 2013; Lai et al., 2015). Following this, the muscle work increased with the accelerated walking speeds. Moreover, Lai et al. (2015) compared changes in fascicular lengths and shortening velocities between walking and running during gait transition speeds; they revealed that changes in fascicular length and shortening velocities in walking were higher than in running. This result suggested that a large muscle force from the ankle plantar flexors was needed during the stance in case of fast walking speeds. Because of this, the requirement of a large muscle force during the stance might affect the Lyapunov exponents of the activation related to forward propulsion (C_2_).

In addition to the maximum Lyapunov exponents in C_2_, the maximum Lyapunov exponents of the activation in Synergy3 and synergy4 might be influenced by the requirement of a large motor output. Synergy3 were constructed by AL, TFL and ERE, and Synergy4 comprised the RF and TA (Figure 3). Both Synergy 3 and Synergy4 were related to the swing leg (60–100% of gait cycle). For normal walking speeds, a simple inverted pendulum model achieves walking without muscle activity during the swing phase (Srinivasan and Ruina, 2006); in addition, the energy cost of the swing leg was found to be small (Gottschall and Kram, 2005). These studies suggest that the swing leg is achieved by small activations of muscles. However, the contributions of the swing leg increase at fast walking speeds, and it has been reported that the metabolic work of swing legs increased at high frequencies (Doke et al., 2005). Moreover, activation of the RF during the swing phase while walking was higher than the activation during running when treadmill speeds were over gait transition speeds (Prilutsky and Gregor, 2001). They also reported average gait transition speed was 2.1 ± 0.2 m/s. This gait speed is too fast for achieving comfortable walking because very fast swing leg is required. Therefore, the muscle activity during the swing phase is exceedingly high in cases of fast walking speeds. We supposed that the maximum Lyapunov exponents of Synergy3 and Synergy4 were high when the swing leg motion was extremely large. Moreover, Synergy5, which was related to leg deceleration, also might be affected by large swing leg motion. Because the swing leg speed is fast during fast walking speeds, a large amount of effort is required to decelerate the swing leg during fast walking speeds. Therefore, the increasing maximum Lyapunov exponents in activations of Synergy3, Synergy4, and Synergy5 at faster walking speeds might relate to fast swing leg motion.

### The Maximum Lyapunov Exponents in the Activation of Muscle Synergies During Slow Walking Speeds

We observed that the maximum Lyapunov exponents of the activations were small at slow walking speeds (Figure 4). This indicates that the maximum Lyapunov exponents of the activations were small at slow walking speeds. Walking speeds have been found to be frequently reduced in individuals with disorders of the CNS (Balasubramanian et al., 2007; Clark et al., 2010; Steele et al., 2015). Moreover, many studies have represented that walking speeds in elderly adults are reduced (Judge et al., 1996; Kerrigan et al., 1998; Lockhart and Liu, 2008). Thus, walking speed is frequently reduced due to the effect of disorders of the CNS or aging. These reductions in walking speed may reflect disabilities of walking. However, we supposed that slow walking speeds may have advantages for the patients who have disorders of the CNS or elderly adults because the maximum Lyapunov exponents of the activations are small at slow walking speeds. It has been revealed that the maximum Lyapunov exponents of kinematic data in individuals with cerebellar ataxia (Hoogkamer et al., 2015), Parkinson disease (Kurz et al., 2010), and a history of falls (Lockhart and Liu, 2008) were higher than those of healthy or young adults. For these individuals, the maximum Lyapunov exponents of the kinematics were high and were needed to stabilize walking. We supposed that one of the strategies for the stabilization of walking was to reduce walking speed. Because the maximum Lyapunov exponents of activation were small at walking speeds, slow walking may be advantageous for the patients with cerebellar ataxia or Parkinson's disease and elderly adults. Therefore, we need to investigate the maximum Lyapunov exponents of activation between healthy adults and elderly or patients with CNS disorders as the future studies.

### The Maximum Floquet Multipliers in the Activation of Muscle Synergies

We observed that the maximum Floquet multipliers of the activations did not depend on the walking speed (Figure 4). It has been revealed that the maximum Floquet multipliers in the trunk are relatively invariant with changes in walking speeds (Kang and Dingwell, 2008). In addition, there were less relationships between the maximum Floquet multipliers and fall-risks (Bruijn et al., 2011). From this evidence, it is thought that the motion of walking is considerably stable in terms of orbital stability. We supposed that the maximum Floquet multipliers of the activations may contribute to periodic walking motions among widely walking speed. Although the maximum Floquet multipliers were invariant with changes in walking speeds, the maximum Floquet multipliers increased due to the effect of added mass (Arellano et al., 2009) or surface perturbations (Sinitksi et al., 2012). These results suggest that the maximum Floquet multipliers are affected by large external forces. Therefore, we might be able to evaluate the orbital stability under the disturbance response rather than at various walking speeds.

We should describe a limitation of present study that evaluating both of Floquet multipliers and Lyapunov exponents. For the deterministic system, both of Lyapunov exponents and Floquet multipliers should be same characteristics. In other words, the Lyapunov exponents should be negative if the Floquet multipliers >1. However, our results did not indicate this characteristics. This discrepancy is one of the limitation in the present study. We considered that discrepancy was derived from the point that the finite-time Lyapunov exponents might reflect the natural variability and intrinsic biological noise in the system. Dingwell and Marin (2006) also referred “the local divergence curves would always exhibit positive divergence reflecting the natural variability and intrinsic biological noise in the system.” Therefore, it is compromised point that we cannot observe the correspondence between the Lyapunov exponents and Floquet multipliers. To estimate the Lyapunov exponent more effectively, we might need to provide appropriate method for computing the finite-time Lyapunov exponents that can be correspond to the Floquet multipliers.

### Similarity of Muscle Synergies

We performed functional sorting of muscle synergies based on the cosine similarities between average muscle synergies and other muscle synergies. In the representative example of Figure 3, the muscle synergy 5 at 7.5 km/h was looks like different from other Synergy 5. However, weighting and activation of this muscle synergy (Synergy 5 at 7.5 km/h) were similar to average muscle synergies among subjects. This indicate that Synergy 5 at 7.5 km/h was similar to common muscle synergy among subjects. Average muscle synergies were indicated in Supplementary Figure 1.

Although there are some ways to perform functional sorting of muscle synergies, we set the present criterion of sorting (*r* of W or C > 0.71) by trial and error to analyse common muscle synergies among subjects. We have tried various criteria for sorting or set various reference muscle synergies (e.g., *r* of W > 0.71, *r* of C > 0.71, or setting the reference muscle synergies as preferred walking speeds). In case we used these criteria, many subject-specific muscle synergies were provided. We cannot investigate the common characteristics of muscle synergies, if there are too many subject-specific muscle synergies. Because of such problems, we set the present criteria (*r* of W or C > 0.71).

## Summary and Conclusion

In summary, this study investigated the maximum Lyapunov exponents and maximum Floquet multipliers in activations of muscle synergies across various walking speeds to identify the local dynamic stability and orbital stability in the activations of muscle synergies that relate to subtasks within a gait cycle. We revealed that the maximum Lyapunov exponents increased with accelerations in walking speeds. Contrary to the maximum Lyapunov exponents, the maximum Floquet multipliers of the activations remained almost constant across the different walking speeds. Although the requirement of a large motor output may cause a great deal of maximum Lyapunov exponents in the activations of muscle synergies, the activations of the muscle synergies were stable in terms of orbital stability. In addition, the increasing rates of the maximum Lyapunov exponents were different among the muscle synergies. Therefore, the local dynamic stability in the muscle synergies might depend on the requirement of motor output related to subtasks within a gait cycle. We concluded that the local dynamic stability in the muscles synergies decreases with accelerations in walking speed. On the other hand, the orbital stability is sustained across walking speeds.

## Data Availability Statement

The datasets analyzed during the current study are available from the corresponding author on reasonable request.

## Author Contributions

BK, SH, TM, and MK: conception and design of the experiments and final approval of the version to be published; BK and SH: collection, analysis and interpretation of the data; BK, SH, and MK: drafting the article or critically revising for important intellectual content.

### Conflict of Interest Statement

The authors declare that the research was conducted in the absence of any commercial or financial relationships that could be construed as a potential conflict of interest.
