# Supplementary material for: Lower Local Dynamic Stability and Invariable Orbital Stability in the Activation of Muscle Synergies in Response to Accelerated Walking Speeds
Source: Front Hum Neurosci. 2018 Dec 11;12:485. doi: 10.3389/fnhum.2018.00485 (PMC6297374; doi:10.3389/fnhum.2018.00485)
Supplement: Supplementary file 1 [file Data_Sheet_1.docx]

Supplementary Material

**Lower local dynamic stability and invariable orbital stability in the activation of muscle synergies in response to accelerated walking speeds**

***Authors:* Benio Kibushi^1,2^, Shota Hagio^2,3^, Toshio Moritani^4^, and Motoki Kouzaki^1*^**

*** *Corresponding Author:***

Motoki Kouzaki, Ph.D.
Laboratory of Neurophysiology, Graduate School of Human and Environmental Studies, Kyoto University
Yoshida-nihonamatsu, Sakyo-ku, Kyoto, Kyoto, Japan 606-8501
Tel. & Fax: +81-75-753-2927
E-mail: [kouzaki.motoki.4x@kyoto-u.ac.jp](mailto:kouzaki.motoki.4x@kyoto-u.ac.jp)


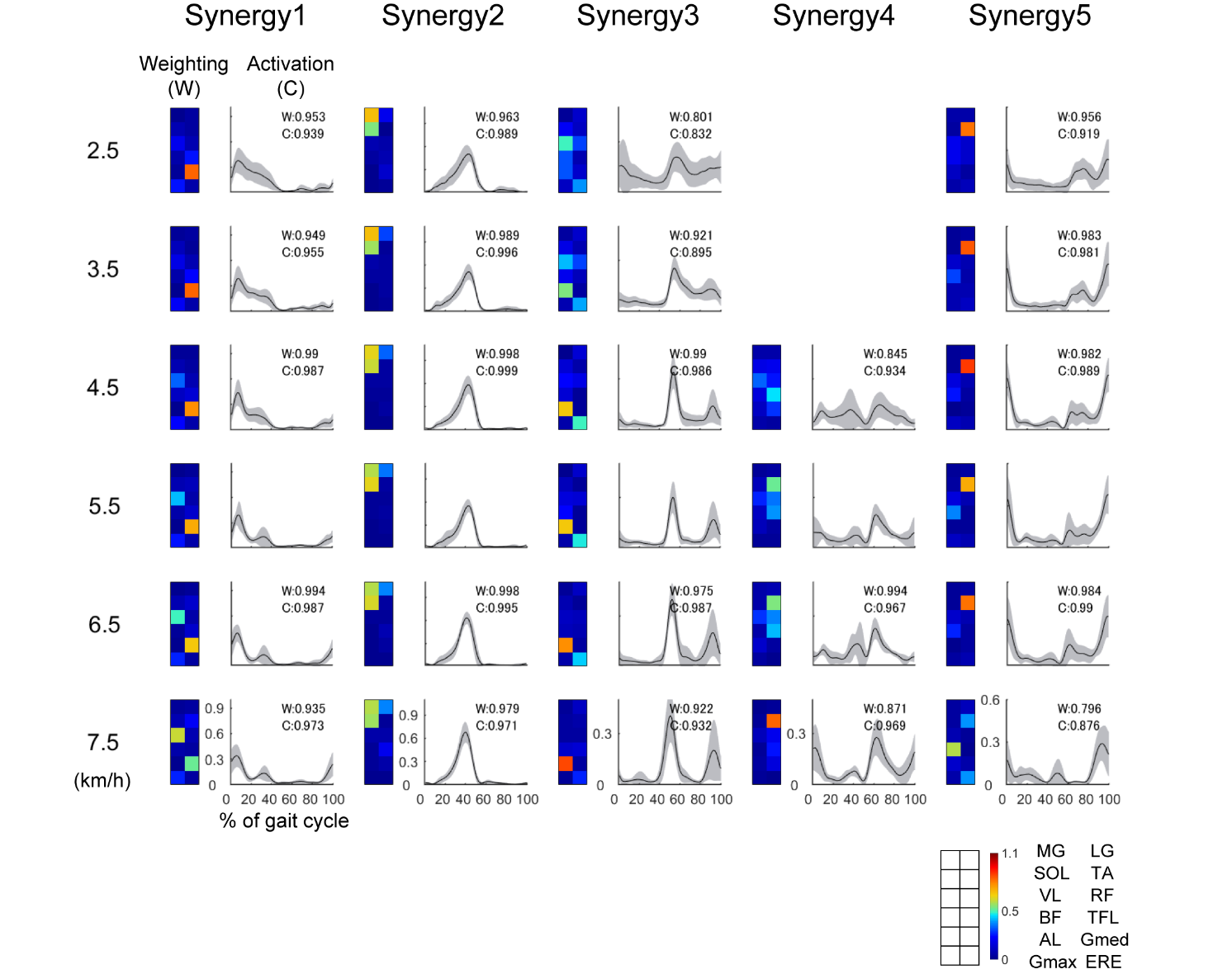


**Supplementary Figure 1.** Average muscle synergies and their activations at 2.5, 3.5, 4.5, 6.7 and 7.5 km/h. The weightings of the muscle synergies (W) are shown as colormaps. Warm color indicates high weightings, and cold color is low weightings. Correspondence between colormap and muscles is described in the lower right of figure. The activations of the muscle synergies (C) within one gait cycle are shown as waveforms. The horizontal axis indicates % of gait cycle, and vertical axis means amplitude of activation. Black lines denote the average activation over 30 gait cycles, and the grey-shaded area denotes the standard deviation of the activation. We performed functional sorting of the muscle synergies by using cosine similarity (Hagio et al., 2015; Kibushi et al., 2018). The functional sorting was performed by grouping the muscle synergies based on the values of cosine similarity compared to that of average muscle synergies at 5.5 km/h. When cosine similarities of W or C were over 0.71 (p<0.01), the muscle synergies were sorted as similar muscle synergies. The values of cosine similarities were indicated at right upper side of plots in the activation. The abbreviations represent the gastrocnemius medialis (MG), gastrocnemius lateralis (LG), soleus (SOL), tibialis anterior (TA), vastus lateralis (VL), rectus femoris (RF), biceps femoris (BF), tensor fasciae latae (TFL), adductor longus (AL), gluteus medius (Gmed), gluteus maximus (Gmax) and erector spinae (ERE).
